# Satellite SST-Based Coral Disease Outbreak Predictions for the Hawaiian Archipelago

**DOI:** 10.3390/rs8020093

**Published:** 2016-01-26

**Authors:** Jamie M. Caldwell, Scott F. Heron, C. Mark Eakin, Megan J. Donahue

**Affiliations:** 1 Hawai’i Institute of Marine Biology, School of Ocean and Earth Science and Technology, University of Hawai’i, Kāne‘ohe, HI 96744, USA; donahuem@hawaii.edu; 2 Coral ReefWatch, U.S. National Oceanic and Atmospheric Administration, College Park, MD 20740, USA; scott.heron@noaa.gov (S.F.H.); mark.eakin@noaa.gov (C.M.E.); 3 Marine Geophysical Laboratory, Physics Department, College of Science, Technology and Engineering, James Cook University, Townsville, QLD 4811, Australia; 4 Global Science and Technology, Inc., Greenbelt, MD 20770, USA

**Keywords:** disease outbreaks, corals, SST metrics, cold snaps, hot snaps, winter condition, MPSA, boosted regression trees, Hawaiian archipelago, models

## Abstract

Predicting wildlife disease risk is essential for effective monitoring and management, especially for geographically expansive ecosystems such as coral reefs in the Hawaiian archipelago. Warming ocean temperature has increased coral disease outbreaks contributing to declines in coral cover worldwide. In this study we investigated seasonal effects of thermal stress on the prevalence of the three most widespread coral diseases in Hawai’i: *Montipora* white syndrome, *Porites* growth anomalies and *Porites* tissue loss syndrome. To predict outbreak likelihood we compared disease prevalence from surveys conducted between 2004 and 2015 from 18 Hawaiian Islands and atolls with biotic (e.g., coral density) and abiotic (satellite-derived sea surface temperature metrics) variables using boosted regression trees. To date, the only coral disease forecast models available were developed for *Acropora* white syndrome on the Great Barrier Reef (GBR). Given the complexities of disease etiology, differences in host demography and environmental conditions across reef regions, it is important to refine and adapt such models for different diseases and geographic regions of interest. Similar to the *Acropora* white syndrome models, anomalously warm conditions were important for predicting *Montipora* white syndrome, possibly due to a relationship between thermal stress and a compromised host immune system. However, coral density and winter conditions were the most important predictors of all three coral diseases in this study, enabling development of a forecasting system that can predict regions of elevated disease risk up to six months before an expected outbreak. Our research indicates satellite-derived systems for forecasting disease outbreaks can be appropriately adapted from the GBR tools and applied for a variety of diseases in a new region. These models can be used to enhance management capacity to prepare for and respond to emerging coral diseases throughout Hawai’i and can be modified for other diseases and regions around the world.

## 1. Introduction

Climate change and associated ocean warming have been linked to increasing frequency and severity of infectious diseases in several economically and ecologically important marine organisms [1,2]. While diseases are an integral component of normally functioning ecosystems, outbreaks can alter population structure, lead to large-scale die-offs and even host extinctions [3,4]. Host-pathogen interactions often shift in response to prolonged or severe environmental disturbance, such as high temperatures or rainfall, which alters interactions in favor of either the host or the pathogen (e.g., promoting pathogen growth while reducing host immune efficacy) [5]. Temperature changes alone can affect host and pathogen behavior, shift pathogen ranges and increase host susceptibility and pathogen virulence [2,5]. For example, a + 1 °C increase in water temperature increases the abundance of pathogenic bacteria *Vibrio harveyi* in the water column and the susceptibility of its abalone host *Haliotis tuerculata* [6]. As periods of anomalously high ocean temperatures occur more frequently, marine disease outbreaks are expected to become more common and more severe.

Links between warming ocean temperatures, increased temperature variability and coral disease outbreaks threaten coral reef ecosystems across the globe. With increasing disease outbreaks and coral bleaching, many corals worldwide are threatened by extinction [7], presenting concerns for food security, livelihoods and shoreline protection for coastal communities [8]. Disease prevalence patterns vary across ocean basins and are strongly correlated with regionally warm temperature anomalies [9]. Previous studies investigating temperature-disease relationships have found that increased ocean temperatures promote lesion and pathogen growth rates [10,11], increase transmission and virulence of pathogens, and reduce coral resistance to pathogens for a variety of coral diseases such as Black Band Disease, Yellow Band Disease and White Plague [1,2,12,13]. While disease outbreaks have been most severe across the Caribbean, coral disease outbreaks have become increasingly common in the Indo-Pacific during the last decade [14–16]. Mild winters and anomalously warm summers have coincided with many of these outbreak events [7,17–19] suggesting an association between broad scale oceanic temperature regimes and disease onset.

Four previous modeling efforts have advanced our ability to predict coral disease outbreaks using remotely sensed sea surface temperature (SST) anomalies. Bruno *et al.* used the weekly SST anomalies metric (WSSTA; the number of weeks in the preceding year at or above +1 °C above the weekly mean), coral cover and long-term *Acropora* white syndrome observations to model outbreak events on the Great Barrier Reef (GBR) [20]. They found that sites with >50% coral cover and more than five anomalously warm weeks were associated with higher disease abundance. Maynard *et al.* also assessed the relationship among white syndrome abundance, coral cover and warm-season high thermal stress using the Mean Positive Summer Anomaly (MPSA) metric in a regression model that predicted outbreak likelihood with >90% accuracy on reefs with >26% coral cover [18]. This study revealed that as temperature stress increased, outbreaks could occur at lower threshold densities.

Heron *et al.* incorporated the magnitude (not just occurrence) of summer anomalies and also included anomalous winter temperature metrics (Winter Condition and Cold Snap) during the year prior to a disease event to predict an outbreak [17]. Their study confirmed the importance of high coral cover (threshold >30%) and anomalously warm summer temperatures for increased risk of *Acropora* white syndrome, but also demonstrated that mild winter temperatures may be equally as important as the two other factors in susceptibility to disease outbreaks. The National Oceanic and Atmospheric Administration (NOAA) now regularly produces a predictive tool for coral disease outbreak risk on the GBR based on these findings [21].

Randall and van Woesik developed a model for white-band disease on *Acropora* in the Caribbean by comparing recent outbreaks with historical and contemporary SST metrics [22]. Their study revealed that the most useful predictors of white-band disease differed depending on the host species, where depth, 30-year rate of temperature change and minimum temperatures were most important for *Acropora palmata* while maximum, minimum and monthly rate of temperature change was most important for *Acropora cervicornis*. These four research efforts set the foundation to develop predictive models for additional temperature-dependent coral diseases in other regions across the globe.

Scientists and managers currently use the Maynard *et al.* and Heron *et al. Acropora* white syndrome models to plan research and conservation efforts in Australia [23]. Remote sensing of environmental conditions associated with disease outbreaks is particularly useful for reef management agencies where time and financial resources are limited, allowing them to better monitor broad geographic regions with low background levels of disease and patchy distribution of outbreaks. Given the complexities of disease etiology, differences in host demography and environmental conditions across reef regions, such models may need to be refined and adapted for different diseases and geographic regions.

Metrics from Heron *et al.* were adapted to produce a complementary, experimental predictive tool for the Hawaiian archipelago [17]. The modifications included determining appropriate SST climatologies, adjusting metric thresholds to represent the new location, and refining the release schedule for seasonal risk outlooks and assessments. However, these metrics and thresholds had, until now, never been quantitatively evaluated using disease observations.

Here, we evaluate the applicability of SST-based metrics describing anomalously warm and cold conditions for forecasting three common coral diseases in Hawai’i: *Montipora* white syndrome, *Porites* growth anomalies and *Porites* tissue loss syndrome. These diseases have caused significant morbidity, reduced fecundity, and often colony mortality in the region’s dominant reef-building corals. *Montipora* white syndrome and *Porites* tissue loss syndrome are characterized by gradual to rapid tissue mortality as the disease progresses across the colony, sometimes killing colonies in less than two weeks [24–26]; *Porites* growth anomalies are chronic, protuberant masses of the coral skeleton that do not cause rapid mortality, but do have a number of deleterious effects on coral including reduced growth, fecundity, fitness and overall survival [27].

*Montipora* (and *Acropora*) white syndrome, *Porites* growth anomalies and *Porites* tissue loss syndrome have been observed to be related to high temperature [26,28], which suggests that these three diseases are good candidates to test thermal stress metrics for forecasting future outbreaks. The pathogenic bacteria that cause white syndromes in *Acropora* and *Montipora* (*Vibrio* spp.) have been observed to alter their disease presentation, decrease time until disease onset and/or shorten the incubation period in higher water temperatures [29,30]. The pathogens that cause *Porites* growth anomalies and tissue loss syndrome are currently unknown, but previous studies have shown that sites with lower thermal stress were associated with higher disease prevalence [26]. To determine outbreak risk in this study, we used boosted regression trees, an ensemble statistical modeling approach that allowed us to identify optimal predictors and their relative importance while minimizing predictive deviance.

## 2. Experimental Section

### 2.1. Field Surveys of Coral Diseases

**Figure 1:**
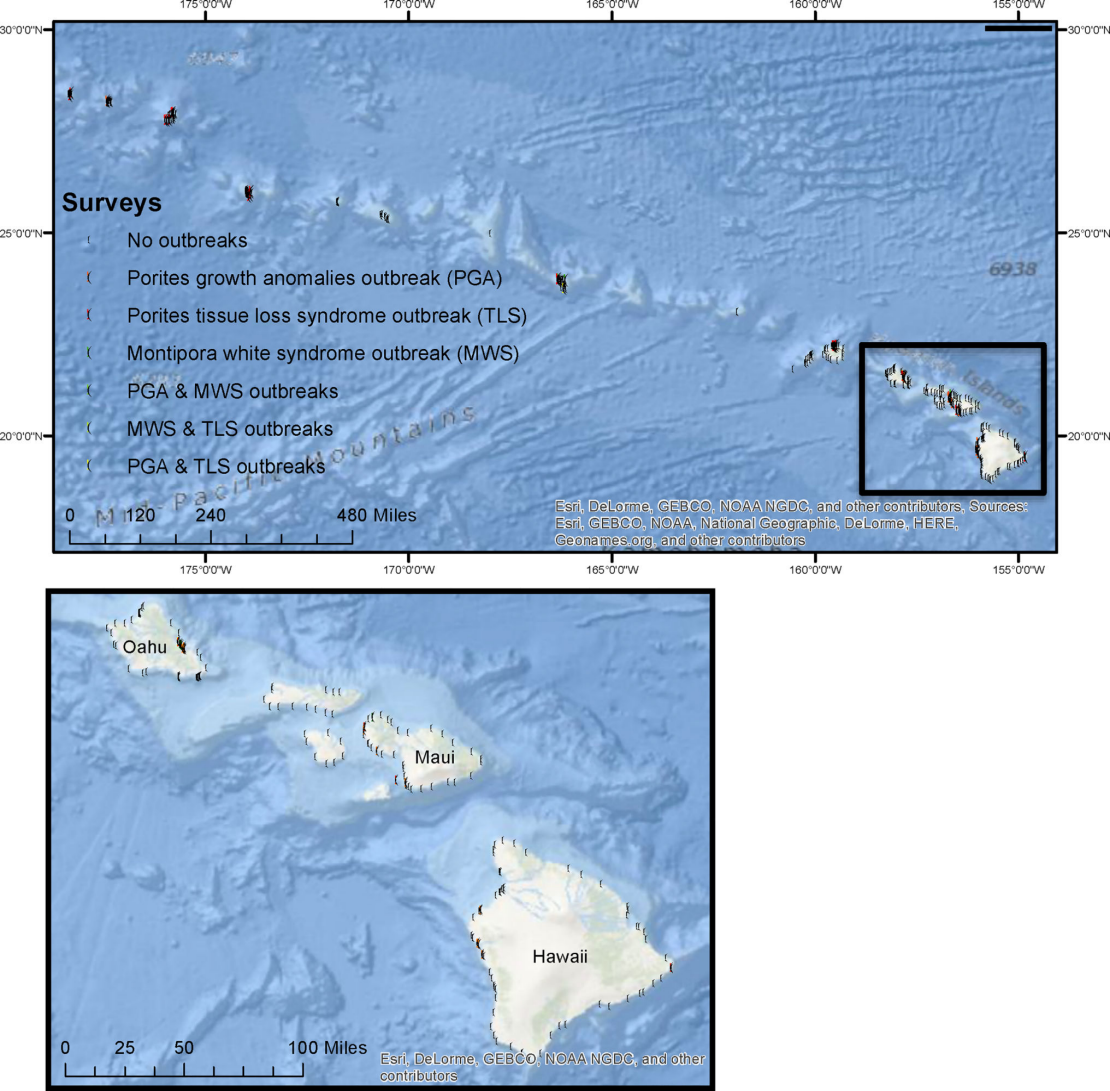
Map of disease surveys in the Hawaiian Survey locations (dots) between 2004 and 2015 were along the extent of the archipelago. Colored dots indicate locations where an outbreak occurred.

We used 789 coral health surveys from the Hawai’i Coral Disease database (HICORDIS) conducted in the Hawaiian archipelago between 2004 and 2015 to explore associations among SST, biotic factors and disease prevalence. Surveys were conducted by ten different research groups on 18 islands and atolls across the ~2800 km Hawaiian archipelago (Figure 1) representing large variations in coral community composition, densities and disease prevalence across nine degrees of latitude (Table 1).

Coral health observations were collected using one of three methods: belt transects, line-intercept and estimated prevalence, across a 1–24.5 m depth range. For the belt transect method, divers recorded health information on all coral colonies within a specified length and width (average length = 20 m, range = 8–50 m; average width = 1 m, range = 1–6 m). In the line-intercept method, divers recorded coral health information for all colonies lying directly beneath 25 m of transect tape. In the estimated prevalence method, divers counted the number of colonies in a subset of a belt transect (average = 10 × 2 m^2^, range = 10 × 2 m^2^ to 10 × 6 m^2^), and counted all diseased colonies within the larger belt transect area (average = 25 × 2 m^2^, range = 25 × 2 m^2^ to 25 × 6 m^2^).

For each survey, we calculated disease prevalence as the number of infected colonies divided by the total number of host colonies (*i.e., Montipora* or *Porites*) observed. For the estimated prevalence method, we extrapolated the total number of host colonies observed for the entire transect surveyed from the abundances counted in the subset region. We excluded surveys that had <20 total coral colony observations from the data because they disproportionately resulted in exceptionally high prevalence values. We calculated densities as the total number of colonies divided by the area surveyed.

### 2.2. Defining Disease Outbreaks

We calculated disease prevalence thresholds by statistically isolating outliers as “outbreaks” (*i.e.*, above average levels of disease prevalence), according to the Heron *et al.* [17] method. Briefly, for each disease, we first isolated the maximum disease prevalence value for each year from 2004 to 2015 and calculated the mean and standard deviation (SD) of the maximum prevalence across years. Any annual values that exceeded the mean plus one SD were replaced with the next highest value from that year and the mean and SD recalculated. This was iteratively repeated until no values were above the threshold. Any values above the final threshold were considered outbreaks.

### 2.3. SST-Based Metrics

To hindcast SST anomalies over the same time period as our disease observations and to develop a tool relevant for forecasting disease risk into the future, we concatenated three SST datasets. We used an updated release (v5.2) of the retrospective Pathfinder SST dataset [31] used in other studies [17] that spanned the period 1985–2012. Daily data were composited to weekly temporal resolution, maintaining the native 1/24° (~4 km) resolution and gap filled following Heron *et al.* [17]. The second dataset was the NOAA/NESDIS Blended 5 km (precisely 0.05°) dataset available in near real-time since March 2013 with daily temporal resolution [32]. This was composited as a weekly average for consistency of temporal resolution with the 4 km dataset. As the 5 km dataset is produced in near real-time, it is the leading option on which to build any future forecasting of disease risk. To bridge the gap between these datasets and to facilitate the bias adjustment between them (ensuring consistency of the time series), the blended 11 km SST predecessor to the 5 km product was used. This product spanned the period February 2009–October 2013 and was also composited to weekly resolution. The three datasets were concatenated by linearly regressing paired weekly values in the temporal overlap and adjusting the relative bias of the 4 km and 11 km values to emulate the 5 km data. This resulted in an internally consistent SST time series at weekly resolution for each survey location and for the period January 1985–June 2015. This covered the period of observations (2004–2015) and allowed development of long-term climatologies used in the calculation of SST metrics.

To examine heat stress preceding each disease survey, we used two SST metrics that have been successfully used to hind/forecast *Acropora* white syndrome on the Great Barrier Reef (Table 1). Mean Positive Summer Anomaly (MPSA) is the average of SST anomalies above the corresponding monthly mean climatology plus one standard deviation (SD), calculated across the three summer months and is expressed as °C [18]. Hot Snap [17] is another indicator of unusually warm conditions experienced primarily during the summer period accounting for both the magnitude and duration of heat stress. The Hot Snap metric uses a single summertime baseline to identify stressful temperature, one SD above the summer mean SST, and integrated the magnitude of temperature anomalies above this baseline through time (units of °C-weeks). Hot Snaps also include positive anomalies outside of the climatological warmest months to include warming prior/subsequent to the three-month summer season.

We also examined two winter metrics that were designed to determine if unusually cold or warm temperatures affect pathogen loading and/or host susceptibility, ultimately increasing risk of disease in the subsequent warmer months as thermal stress accumulates [17]. Cold Snap [17] combine the magnitude and duration of wintertime cold stress by integrating temperatures that are one SD below the mean winter SST (climatological mean across three coldest months of the year). The Cold Snap metric consists of only negative values and is calculated across the nine months preceding the summer. The Winter Condition [17] complements the Cold Snap metric to provide an overall measure of winter pre-conditioning throughout the year. The Winter Condition consists of positive and negative anomalies accumulated about the mean winter temperature through the defined three-month winter period and also incorporates additional non-winter cool periods when temperatures are below one winter SD above the winter mean.

While the temperature profile in the ocean varies with depth, temperature anomalies from the surface provide an effective estimate of anomalies to at least a few tens of meters as well as at the surface [17]. As each of these metrics was derived using temperature anomalies, they are indicative of conditions experienced at the depth of corals. However, depth may contribute significant information to the models, so it is independently included as a model parameter and as an interactive term with other SST metrics. We calculated all SST-based metrics for each pixel containing a disease survey using the concatenated SST time series.

### 2.4. Determining Outbreak Risk

To assess the relationship among SST metrics, biotic factors (e.g., host density) and disease prevalence, we used boosted regression trees. Boosted regression trees (BRTs) fit response variables (*i.e.*, disease prevalence) to their predictors through recursive binary splits in an additive fashion in which simple regression trees are combined using a boosting algorithm (an algorithm to reduce deviance at each iteration) to improve predictive performance [33]. BRTs are models built in a stage-wise fashion using machine-learning techniques to minimize predictive error at each stage of model building and have superior predictive performance compared to other modeling techniques [33–35].

Three parameters need to be defined to optimize model performance in BRTs: tree complexity, learning rate, and bag fraction. Tree complexity (*tc*) controls the number of nodes in a tree based on whether interactions are fitted. Learning rate (*lr*) reduces the contribution of each tree as it is added to the model. Together, tree complexity and learning rate determine the number of trees required for optimal predictive power. Number of trees (*nt*) is the number of consecutive trees used to build a BRT. The optimal *nt* indicates the point just before the model begins to overfit the data. Using an *nt* beyond the optimal *nt* may increase predictive deviance. Stochasticity adds accuracy and reduces over fitting and is controlled in BRTs through a bag fraction, which uses a bootstrapped subset of data to fit each new tree. A bag fraction of 0.5 means that at each iteration, individual trees are fitted with 50% of the data that are drawn at random, without replacement, from the dataset.

To determine the optimal values of learning rate, tree complexity and bag fraction, we examined the cross-validation deviance over *lr* values of 0.01, 0.001, 0.005, *tc* values of 1–5 and bag fraction values of 0.1, 0.5, 0.75. Cross validation is a technique for evaluating the model using withheld portions of the data and the cross-validation deviance measures how much the predicted values based on the non-withheld data differ from the observations from the withheld data [33]. We ran all possible combinations using *gbm.step* in the *gbm* package in R statistical program version 3.3.1 [36] with 10-fold cross-validation in each model run and selected the three-parameter combination that produced the lowest cross-validation deviance to produce the optimal BRT. We further optimized the final BRT model by removing redundant, non-informative predictor variables selected through *gbm.simplify*, a method analogous to backwards selection in regression.

For each disease we developed hierarchical BRT models to account for zero-inflated data. The majority of surveys (50%–91% across the three diseases) had no diseased colonies (*i.e.*, a prevalence of zero). We accounted for the zero-inflated data distribution using an approach similar to Cappelle *et al.* [34], where we combined two BRT models: a presence-absence model assuming a Bernoulli response distribution and a prevalence-if-present model using a Gaussian response distribution on square-root transformed prevalence data. The final prevalence prediction was calculated as the probability of disease presence given by the presence-absence model (0 or 1) multiplied by the predicted prevalence given by the prevalence-if-present model.

To test predictive accuracy, we created all models using the parameters described above on a training dataset consisting of a randomly selected 75% subset of surveys and used the withheld 25% of surveys (test data) for independent validation of the models. To evaluate the performance of each BRT individually, we calculated the cross-validation (CV) deviance and the standard error of CV deviance (se). Here, we used CV deviance as a measure of each model’s ability to explain the withheld data in the training dataset.

To determine model performance of the hierarchical BRTs, we calculated the area under the receiver operating characteristic curve (AUC) and model deviance for each hierarchical model. To quantitatively measure goodness-of-fit, we used AUC values, which provide a metric for how well the model distinguishes presences and absences by comparing rates of true positives and false positives. A model that is unable to assign presences more often than random is indicated by an AUC value of 0.5 whereas a model that always assigns presences with a higher probability than absences (perfect fit) is indicated by an AUC value of 1.0. The percentage of test data explained by the model is called deviance (D). We calculated D as the average difference between true observations of disease prevalence in the test dataset and predicted estimates of disease prevalence made by the model.

## 3. Results and Discussion

### 3.1. Defining Disease Outbreaks

Using the method described in Heron *et al.* [17], we defined the occurrence of a disease outbreak for *Montipora* white syndrome, *Porites* growth anomalies and *Porites* tissue loss syndrome in Hawai’i as any survey where prevalence values exceeded 8.5%, 45.5% and 16.2% respectively. According to these thresholds, outbreaks of *Montipora* white syndrome were recorded in 10 surveys (1.3%), outbreaks of *Porites* growth anomalies were recorded in 26 surveys (3.3%) and outbreaks of *Porites* tissue loss syndrome were recorded in 43 surveys (5.4%); there were 789 surveys in total for each disease. Mean prevalence values previously reported for *Montipora* white syndrome and tissue loss syndrome in Hawai’i ranged from 0% to 1% and increased several-fold during outbreak events, previously reported as high as 35.95% and 29.17% respectively [26,37]. In contrast, mean prevalence values previously reported for *Porites* growth anomalies across the Indo-Pacific (including Hawai’i) ranged from 0% to 13.7% illustrating relatively high endemic levels of disease prevalence despite dynamic changes in their presence during the summer and fall [26,37–39]. The large size of this dataset allowed us to define regionally specific outbreak thresholds for the first time. As with all studies in wildlife disease ecology, these thresholds should neither be considered an exact or fixed threshold. However, they provided a meaningful metric to distinguish locations with normal versus above average disease prevalence.

**Figure 2:**
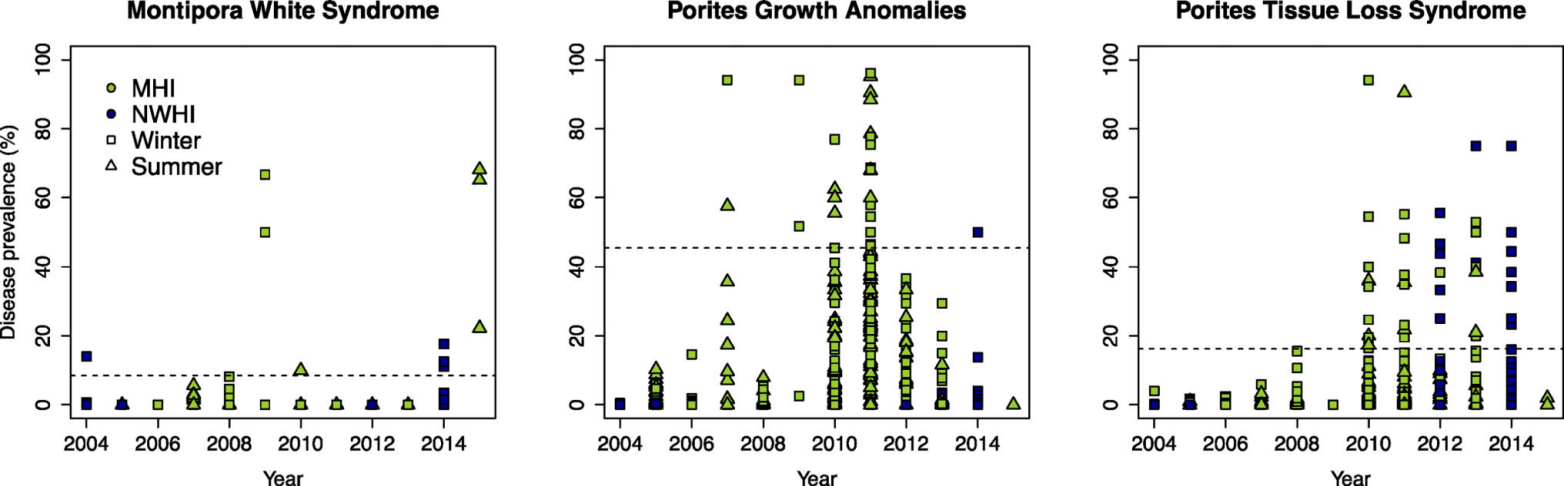
Disease prevalence by disease, year, season and region. of *Montipora* white syndrome, *Porites* growth anomalies and *Porites* tissue loss syndrome prevalence through time. Dashed horizontal lines represent outbreak thresholds determined by the iterative analysis described in the methods. MHI: Main Hawaiian Islands; NWHI: Northwestern Hawaiian Islands. Winter includes surveys conducted in November–April; summer includes surveys conducted in May–October.

Many coral disease outbreaks between 2004 and 2015 occurred clustered in space and time (Figures 1 and 2). The most severe outbreaks for all three diseases occurred in the Main Hawaiian Islands. *Montipora* white syndrome outbreaks primarily occurred during the winter of 2009/2010 and 2015 on Oahu and in 2014 on Maui. Given the importance of host density for *Montipora* white syndrome transmission [24], the high number of susceptible hosts at these specific locations may partially explain this spatial pattern. The majority of outbreaks for *Porites* growth anomalies and tissue loss syndrome also occurred between 2010 and 2015, with the most severe events in 2010 and 2011. Thirteen *Porites* growth anomalies outbreaks (48%) were recorded around the island of O’ahu with six outbreaks (22%) recorded around Hawai’i Island. The spatial clustering of *Porites* growth anomalies around Hawai’i may be partially explained by the correlation between disease prevalence and high host densities whereas the spatial clusters of outbreaks on O’ahu may be explained by the correlation between disease prevalence and large human populations sizes [38]. Ten *Porites* tissue loss syndrome outbreaks (29%) also occurred on O’ahu. Prior studies have shown a positive correlation between *Porites* tissue loss syndrome and coral cover/host density [26,28]. *Porites* is the dominant coral genus on all of the Main Hawaiian Islands, including O’ahu [35], which suggests there are additional factors driving tissue loss syndrome prevalence that are currently unknown.

### 3.2. Determining Outbreak Risk

**Figure 3:**
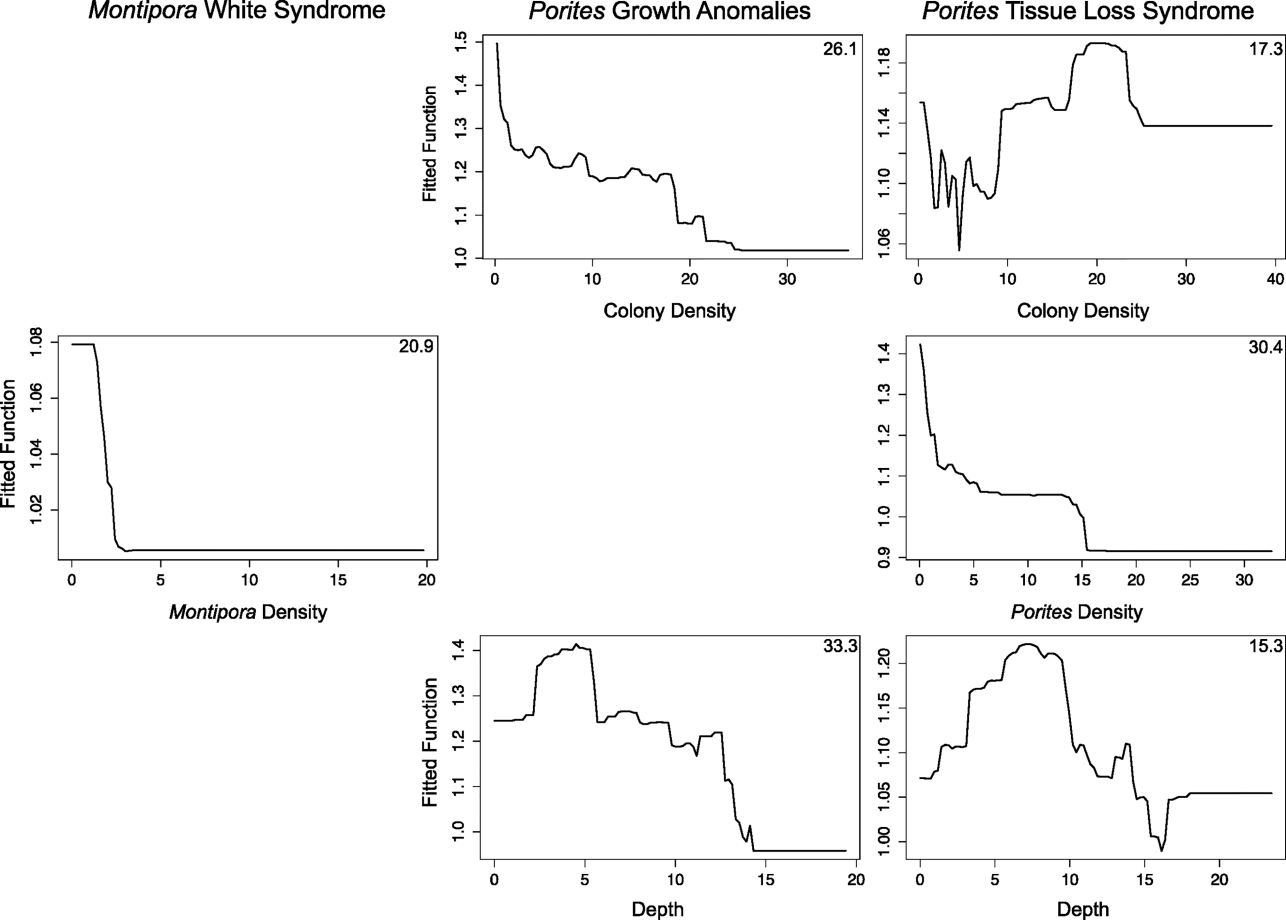

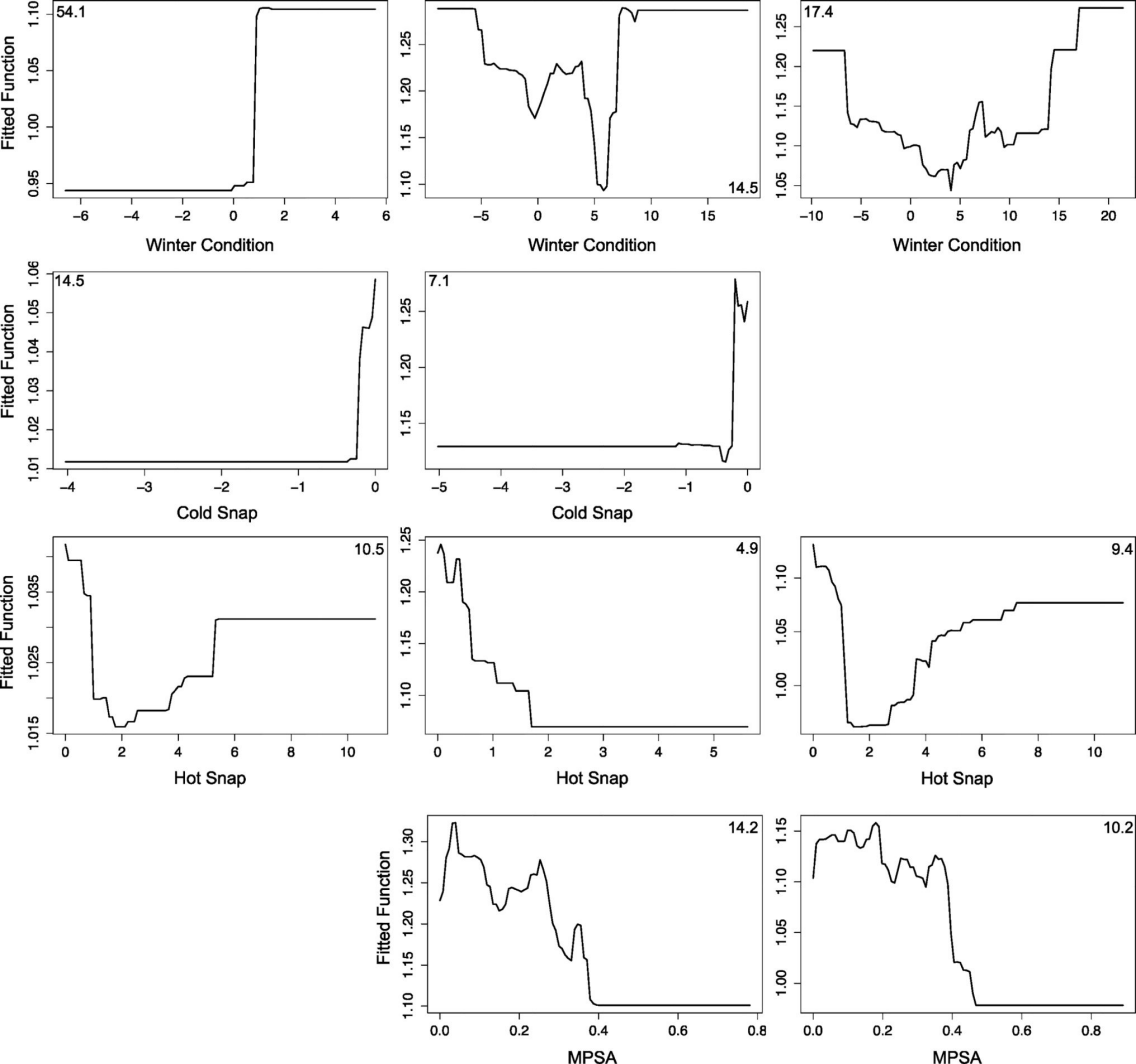
Partial dependence plots relating coral disease prevalence to demographic and thermal predictor variables for prevalence-if-present models. Plots show the probability of disease prevalence across a range of values for the predictor variable, while accounting for the average effects of all other variables in the model. Models were developed with a randomly chosen 75% of the dataset and were tested using the 25% withheld. Relative influence of each predictor variable is shown as a percentage in a corner of each graph. We did not incorporate non-informative predictors, which were determined using the R function *gmb.simplify*, and therefore we do not show partial dependence plots for those variables (blank spaces). Host density is specified by genus (*i.e., Montipora* or *Porites* density). MPSA is Mean Positive Summer Anomaly.

We used hierarchical BRT models to retrospectively predict coral disease prevalence. Optimal parameters for these models ranged from 750 to 3500 trees, tree complexities of 3–5, learning rates of 0.001 or 0.005 and a bag fraction of 0.75 (Table 2). Model cross-validation deviances for the training data ranged from 0.048 to 1.213 (Table 2). Cross-validation deviances closer to zero indicate greater predictive accuracy. AUC values ranged from 0.67 to 0.85. Large AUC values indicate higher performance models. All models predicted disease prevalence better than random (*i.e.*, AUC > 0.5) but the model for *Porites* growth anomalies performed best in the independent validation tests using the test dataset with an AUC value of 0.85 (Table 2). Coral distribution (*i.e., Montipora* density, *Porites* density and/or colony density),Winter Condition and a metric of summer thermal stress (*i.e.*, MPSA and/or Hot Snap) were important for all models; however, relative contributions differed among models (Figure 3). For example, the relative influence of Winter Condition in the *Montipora* white syndrome model was 54.1% compared to only 14.5% in the *Porites* growth anomalies model suggesting that Winter Condition plays a more important role in disease dynamics of *Montipora* white syndrome (Figure 3). We show the relative influence of each predictor variable and the relationship between the fitted model functions and predictor variables in Figure 3.

#### 3.2.1. *Montipora* White Syndrome

Winter Condition, Cold Snap, Hot Snap and host density were the most informative predictors of *Montipora* white syndrome prevalence (Figure 3); all other predictor variables were determined non-informative. The mean predictive accuracy for *Montipora* white syndrome (AUC) was 0.70 and explained 30% of the deviance using the test data (Table 2). The Winter Condition metric had the highest relative influence in the model, highlighting a relationship between mild Winter Conditions (>0.85 °C-weeks) and higher disease prevalence. This relationship is similar to the relationship between winter temperatures and *Acropora* white syndromes on the Great Barrier Reef where Winter Conditions of 2.5–6.5 °C-weeks were associated with higher disease abundance [17]. Also consistent with *Acropora* white syndrome on the GBR [17], few or no Cold Snaps were associated with high disease prevalence, possibly suggesting the pathogenic bacteria that cause *Montipora* white syndrome may have a lower likelihood of survival during very cold winters.

The model results indicated unexpected relationships between disease prevalence and Hot Snaps and host (*i.e., Montipora*) density. The Hot Snap metric had a non-linear relationship with disease prevalence. Similar to *Acropora* white syndrome, there was an association between Hot Snaps above 2 °C-weeks and higher disease prevalence. In contrast to *Acropora* white syndrome however, high disease prevalence was also associated with Hot Snaps less than 1.3 °C-weeks. This relationship may help explain why several *Montipora* white syndrome outbreaks have occurred during winter months in Hawai’i (Figure 2). However additional factors not included in the study may be needed to better understand the seasonality of *Montipora* white syndrome such as increased rainfall and associated runoff. Surprisingly, disease prevalence increased as host density decreased with the highest disease prevalence at sites with <2.7 colonies · m^−2^. More studies are needed to further examine this relationship, however, because this result may reflect host densities in locations with persistent severe outbreaks such as Kāne’ohe Bay, O’ahu, rather than real threshold host densities needed for disease establishment.

#### 3.2.2. *Porites* Growth Anomalies

The model developed for *Porites* growth anomalies prevalence had the highest predictive performance where depth and colony density were the strongest drivers of disease prevalence (Figure 3). The AUC value for the hierarchical *Porites* growth anomalies model was 0.85 and explained 41% of the deviance using the test data (Table 2). Given the high relative influence of site level characteristics in predicting disease prevalence, thermal stress is most likely to increase disease prevalence at sites in specific depth and host density ranges. Spatially explicit maps of such site level characteristics can be developed (and subsequently updated) to complement thermal risk assessments. There were negative relationships between both depth and colony density and disease prevalence, where shallower coral communities (<12.7 m) and lower colony densities were associated with higher disease prevalence. Although the relationship between shallower depths and *Porites* growth anomalies prevalence is currently unclear, one previous study determined that UV radiation does not explain this relationship [27]. Within Kāne’ohe Bay, O’ahu,Williams *et al.* [28] found a negative relationship between coral cover and *Porites* growth anomalies prevalence; our study includes the Williams *et al.* dataset and suggests a similar relationship may exist across the entire archipelago. One possible explanation for the negative relationship between colony density and disease is that higher colony density often reflects a community of very small colonies, and *Porites* growth anomalies are more common on larger corals [39].

Winter Condition, MPSA and Hot Snap significantly contributed to predictive accuracy of the *Porites* growth anomalies model (Figure 3). A bi-modal relationship exists between Winter Condition and disease prevalence where cold (<−5 °C-weeks) and warm (>8.8 °C-weeks) conditions were associated with higher disease prevalence. There was a negative relationship between disease prevalence and both Mean Positive Summer Anomaly (MPSA) and Hot Snap, indicating high thermal stress is associated with lower disease prevalence. This combination of colder winters and mild summers compliments a prior study that found a negative association between *Porites* growth anomalies and average occurrence of anomalously warm temperature (WSSTA) over the four years prior to a survey [26]. This combination may also help explain the shorter-term seasonality associated with increased growth anomaly density and subsequent growth in the fall [27].

#### 3.2.3. *Porites* Tissue Loss Syndrome

Host and coral density, depth, Winter Condition, MPSA and Hot Snap were important for predicting *Porites* tissue loss syndrome prevalence (Figure 3). The mean predictive accuracy for *Porites* tissue loss syndrome (AUC) was 0.67 and explained 44% of the deviance using the test data (Table 2). There was an interaction between host density, coral density and disease prevalence, in which sites with high coral density but lower host density were associated with the highest disease prevalence values. Disease prevalence was also highest in shallower water (<7 m). The Winter Condition metric shows a negative association between disease prevalence and cold temperature (<0.3 °C-weeks) and a positive relationship between disease prevalence and warm temperature (>4.4 °C-weeks). The Hot Snap metric above 2 °C-weeks supports a positive relationship between thermal stress and disease prevalence, also shown in the MPSA, however, the Hot Snap metric <1.6 °C-weeks contradicts this pattern. Our conclusion is that our data are likely missing an important predictor variable for explaining tissue loss prevalence such as additional metrics of coral distribution, water quality, or oceanographic conditions.

Very little is currently known about the disease etiology of *Porites* tissue loss syndrome and the role of thermal conditions in influencing disease dynamics. The results here suggest extreme thermal conditions in the winter may contribute to coral susceptibility to disease, but more information is needed to build a robust predictive model. The bimodal relationship between thermal stress and disease could support one or two complimentary hypotheses about winter pre-conditioning and disease prevalence: (1) during anomalously warm winters pathogens do not undergo winter mortalities leading to higher densities of pathogenic organisms in the water column; and (2) thermal stress prior to the summer contributes to compromised host immune systems. Both hypotheses require further exploration.

### 3.3. Forecasting Disease Risk

To accurately predict coral disease risk in Hawai’i, information on both coral distribution and thermal stress is needed. For all three diseases modeled in this study, thermal stress was only important for predicting disease risk in communities within a specific range of colony densities. A fundamental principle of epidemiological theory is that there is a minimum population threshold for pathogen invasion and a critical community size required for disease persistence for diseases with density dependent transmission [40]. While exact thresholds in wildlife populations are difficult to determine, identifying host distribution ranges around these thresholds, as we did here, can improve our understanding of disease dynamics [41] and therefore ability to forecast future outbreak events. For non-infectious diseases, where pathogens are endemic in the population and infection results from an imbalance in the host–pathogen relationship, variables besides host density may be important determinants of disease prevalence [42]. For example, in this study we found that depth was an important determinant of disease prevalence for *Porites* growth anomalies and tissue loss syndrome. Depth may be an important predictor of coral disease in Hawai’i because shallower sites are often exposed to greater environmental stress such as nutrient runoff, pollution, salinity changes, increased UV radiation and destructive human activities (e.g., spearfishing). Incorporating size-frequency distributions into future models may also improve predictive accuracy as disease susceptibility often varies with size and/or age structure, such as in the relationship between the fungal pathogen *Aspergillus sydowii* and its sea fan coral host *Gorgonia ventalina* [43].

Winter Conditions were strong predictors of all three coral diseases, highlighting the importance of winter pre-conditioning of hosts and pathogens for disease onset and making it possible to evaluate disease risk several months before an expected outbreak. Warm winter temperature was an indicator of increased disease risk for all three diseases, whereas cold winter temperatures appeared to lower the risk for *Montipora* white syndrome, but not for *Porites* growth anomalies or tissue loss syndrome. High thermal stress in the summer indicated an increased likelihood of *Montipora* white syndrome and *Porites* tissue loss syndrome; however, more information is needed to better understand these relationships at lower levels of thermal stress (*i.e.*, Hot Snaps <2 °C-weeks) given the nonlinear relationships displayed in partial dependence plots (Figure 3).

### 3.4. Uncertainties, Errors and Accuracies

Modeling complex host–pathogen–environment relationships has led to interesting insights into the relationships between coral disease, host distributions, and thermal stress; however, many aspects of how environmental stressors drive coral diseases still remain uncertain. For all three diseases modeled here, the presence-absence models had lower predictive accuracy than the prevalence-if-present models (indicated by the several-fold higher cross-validation deviances). The lower predictive accuracy of the presence-absence models suggests more information is needed to differentiate sites with and without disease. Given that low background levels of disease are natural in all populations, environmental conditions may not differ between places with no visible disease and those with very low levels of disease prevalence. While our results suggest these models can provide an early and good first approximation of expected levels of disease prevalence prior to a disease event, additional environmental information could improve model accuracy.

Several studies have shown relationships between water quality (e.g., turbidity, chlorophyll *a* concentration, sedimentation, nutrient pollution) and disease prevalence [28,44–46] and incorporating such predictor variables could improve disease predictions. Remotely sensed ocean color metrics may provide a useful tool to account for broad scale variation in water quality. However, ocean color metrics require further development and validation for shallow, coastal habitats and would need surface-measured data to validate and/or complement such a study. The relationships with temperature identified here for three diseases can provide the basis for the development and delivery of monitoring tools that can help managers now and can subsequently guide further evaluation of temperature-disease relationships along with those of other environmental parameters.

## 4. Conclusions

Coral diseases arise from complex interactions between host, pathogens and their environment. Climate change is altering these interactions and thus increasing the magnitude and severity of outbreaks worldwide. Modeling efforts such as the one described here help elucidate environmental drivers of disease and may provide risk assessments for future disease outbreaks months before an expected event. Using a combination of methods used for forecasting *Acropora* white syndrome outbreaks on the Great Barrier Reef and white-band disease on *Acropora* in the Caribbean [17,22], we were able to develop separate predictive models for *Montipora* white syndrome, *Porites* growth anomalies, and *Porites* tissue loss syndrome in Hawai’i using boosted regression trees. Our research highlights the need for disease- and regionally-specific models given the differences among the models for each host–pathogen relationship and between models developed for Hawai’i and Australia. One reason seasonal thermal stress may be less influential for coral diseases in Hawai’i compared to the Great Barrier Reef is that SST stress is ameliorated through flushing of near shore waters with deep oceanic water found close to shore and through high wave energy found at many locations in Hawai’i [47].

As the frequency of extreme temperature events continues to increase in Hawai’i, forecasting models of temperature-driven diseases will become increasingly useful to resource managers and scientists alike. Before 2014, the Main Hawaiian Islands had only experienced one severe thermal stress event that resulted in mass coral bleaching [48]. Now mass bleaching events have occurred in two consecutive years as a result of thermal stress [49,50]. This study has confirmed that Coral Reef Watch’s Experimental Coral Disease Outbreak Risk product for the Hawaiian archipelago [51] provides valuable insights that can be used by local managers, but the addition of other biotic and abiotic factors could increase predictive accuracy of the forecasts, which currently still produce some false positives and negatives. In addition to the SST metrics and biotic factors used in these models, other biotic and abiotic factors such chlorophyll *a* concentration should be assessed to better understand coral susceptibility to disease and increase model predictive capabilities. The models we developed for *Montipora* white syndrome, *Porites* growth anomalies and *Porites* tissue loss syndrome can improve the capacity of local managers to prepare for and respond to disease outbreaks in Hawai’i several months before an expected outbreak event. This may be especially useful for monitoring remote locations such as coral reefs in the Papahānaumokuākea Marine National Monument.

## Figures and Tables

**Table 1:** Host distribution and environmental predictor variables used in boosted regression trees.

Variable	Type	Description and Unit	Min	Max
Total coral abundance	Biotic	Number of colonies/survey	20	2633
Total coral density	Biotic	Number of colonies/m^2^	0.15	52.4
*Porites* density	Biotic	Number of colonies/m^2^	0.02	9.49
*Montipora* density	Biotic	Number of colonies/m^2^	0.02	8.4
Depth	Abiotic	Meters below sea surface	<1	24.5
Winter Condition	Abiotic	Accumulation of positive and negative thermal anomalies; °C-weeks	−10.162	21.465
Cold Snap	Abiotic	Magnitude and duration of cold stress; °C-weeks	−5.0255	0
MPSA	Abiotic	Mean number of degree heating days in summer; °C	0	0.78077
Hot Snap	Abiotic	Magnitude and duration of heat stress; °C-weeks	0	11.02

**Table 2:** Optimal setting and predictive performance of boosted regression tree analyses for three coral diseases.

Coral Disease	Model	*nt*	*tc*	*lr*	bf	cv dev	se	AUC	D
*Montipora* white syndrome	PA	3500	5	0.001	0.75	0.262	0.036	0.70	0.30
	PIP	1400	4	0.001	0.75	0.113	0.086		

*Porites* growth anomalies	PA	1350	4	0.005	0.75	1.061	0.031	0.85	0.41
	PIP	1900	5	0.005	0.75	0.048	0.005		

*Porites* tissue loss	PA	750	3	0.005	0.75	1.213	0.027	0.67	0.44
	PIP	2700	4	0.005	0.75	0.33	0.004		

Models: PA: Presence-Absence; PIP: Prevalence-If-Present; *nt* number of trees; *tc* tree complexity; *lr* learning rate; bf: bag fraction; cv dev: cross-validation deviance; se: standard error; AUC: area under the operating curve; D: predictive deviance of the final BRT model. Large AUC values indicate higher performance models.
